# Genetic mapping and molecular marker development for white flesh color in tomato

**DOI:** 10.3389/fpls.2024.1459013

**Published:** 2024-09-03

**Authors:** Jie Liu, Xiaoxue Fang, Fangjie Yu, Chengfeng Zhang, Pengfei Fan, Ningdong Wang, Qiao Shao, Ning Gan, Xiaolong Lv, Bo Ouyang, Mingfang Zhang, Xinsheng Wu, Nanqiao Liao

**Affiliations:** ^1^ Department of Molecular Assistant Breeding, Weimeng Seed Co. Ltd., Ningbo, China; ^2^ Key Laboratory of digital seed industry of watermelon, melon & cabbage, Ministry of Agriculture and Rural Areas, Ningbo, China; ^3^ Laboratory of Germplasm Innovation and Molecular Breeding, College of Agriculture and Biotechnology, Zhejiang University, Hangzhou, China; ^4^ Department of Vegetable Science, College of Horticultural and Forest Sciences, Huazhong Agricultural University, Wuhan, China

**Keywords:** tomato, white flesh, gene mapping, molecular breeding, fruit color

## Abstract

**Introduction:**

Fruit color significantly influences the quality of horticultural crops, which affects phytochemical diversity and consumer preferences. Despite its importance, the genetic basis of the white-colored fruit in tomatoes remains poorly understood.

**Methods:**

In this study, we demonstrate that white-fleshed tomato varieties accumulate fewer carotenoids than yellow-fleshed varieties. We developed various segregating populations by hybridizing red, yellow, and white fruit tomato cultivars.

**Results:**

Genetic analysis revealed that the white fruit color trait is controlled by a single gene that dominates both red and yellow fruits. Bulk segregant RNA sequencing provided a preliminary map of a 3.17 Mb region on chromosome 3 associated with the white color trait. Based on kompetitive allele-specific PCR (KASP) markers, we narrowed the candidate gene region to 819 kb. Within this region, we identified a 4906-bp sequence absence variation near Phytoene Synthase 1 (SlPSY1) specific to white-colored tomatoes. Genotyping of the progeny and natural populations using a single nucleotide polymorphism adjacent to this absence of variation confirmed its key role in white fruit formation.

**Discussion:**

Collectively, our findings provide insights into white fruit trait formation in tomatoes, enabling tomato breeders to precisely introduce white fruit traits for commercial exploitation.

## 1 Introduction

Tomatoes (*Solanum lycopersicum* L.) are among the most important vegetables globally. Annual production of fresh tomatoes is expected to reach approximately 254 million tons by 2022 (https://www.fao.org/). Tomatoes are nutritionally well balanced and rich in vitamins and functional pigments, and are important in global food security and nutrition (Chen et al., 2023). The attractiveness of fresh color is a key breeding goal that greatly affects consumer preferences and is determined by pigments in the peel and pericarp (Lin et al., 2014). Plants produce these pigments in flowers and fruits primarily to attract pollinators and seed dispersers (Ballester et al., 2010; Yang et al., 2023). Pigments contribute to both the aesthetic appeal and nutritional benefits of fruits and vegetables favored by consumers. The main pigments in tomatoes include chlorophylls, carotenoids, and flavonoids, which contribute to the red, orange, yellow, green, light green, and white flesh (Li and Yuan, 2013; Kabelka et al., 2004).

Fruit color has been studied for decades as an attractive attribute of horticultural crops. Major genes or quantitative trait loci (QTLs) that influence flesh color have been identified in crops, such as watermelon, melon, cucumber, and peppers. For instance, The *ClLCYB* gene encoding lycopene beta-cyclase, which is responsible for the red flesh color in watermelon, has been mapped to chromosome 4 (Liu et al., 2014). The abundance of the protein is inversely correlated with lycopene accumulation (Zhang et al., 2020). Similarly, the golden flesh trait in watermelons is determined by *ClPSY1* (*Phytoene synthase 1*) on chromosome 4 (Liu et al., 2021), whereas scarlet red flesh color (*Y^scr^*) involves glycine-rich cell wall proteins encoded by candidate genes on chromosome 6 (Li et al., 2020). The plastid lipid-associated protein Cla97C10G185970 likely influences the pale green flesh color in watermelons (Pei et al., 2021). In melon, the *CmOr* gene regulates β-carotene accumulation (Tzuri et al., 2015). The *CmPPR1* gene for white flesh color has been mapped to chromosome 8 (Galpaz et al., 2018). In cucumber, the *ORE* gene on chromosome 3DS is pivotal for β-carotene accumulation (Bo et al., 2011), whereas a single recessive gene (*yf*) on chromosome 7 controls the light yellow flesh color (Lu et al., 2015). In chili pepper, QTLs pc8.1 and pc10.1 influence chlorophyll levels in green-ripe fruits (Brand et al., 2011, 2014), with *CaGLK2* being identified as a candidate gene for pc10.1, which affects chloroplast development, similar to *SlGLK2* from tomato (Brand et al., 2014).

Several major genes associated with flesh color have been identified in tomatoes. The findings have clarified the structural and regulatory pathways that influence mature fruit color. *PSY1* encodes phytoene synthase, a key enzyme in carotenoid biosynthesis that catalyzes the condensation of two geranylgeranyl diphosphate molecules to form 15-cis-phytoene, which is the initial step in carotenoid production (Zhou et al., 2022). Mutants in *PSY1*, such as *r* (yellow flesh) and *r^y^*, result in mature fruits with pale yellow flesh and yellow skin (Fray and Grierson, 1993). Mutation of the carotenoid isomerase gene (*CRTISO*) leads to a significant decrease in carotenoid components downstream of tetra-cis-lycopene, ultimately producing an orange fruit phenotype (Isaacson et al., 2002). *SlMYB12* encodes a crucial transcription factor that regulates naringin chalcone biosynthesis in tomatoes. Deletion of this gene results in a colorless epidermis due to flavonoid deficiency, although the flesh remains red, giving the fruit a pink appearance (Ballester et al., 2010; Zhu et al., 2018). Chlorophyll influences tomato coloration. Mutations in *STAY-GREEN* (*SGR*) inhibit chlorophyll degradation during ripening (Pereira-Castro and Moreira, 2021), leading to the accumulation of lycopene and brown-colored fruits (Barry et al., 2008). A CRISPR/Cas9-mediated multiplex gene editing system was used to successfully generate differently colored fruits of tomato lines from red-fruiting materials by editing three fruit-color-related genes (*PSY1*, *MYB12*, and *SGR1*) (Yang et al., 2023).

Diverse fruit color patterns are favored by both breeders and consumers. White flesh tomato varieties, which harbor natural mutations commonly used in tomato breeding, are particularly attractive because of their distinct fruit color. Here, a series of genetic mappings and molecular analyses of white flesh traits in tomatoes were performed. The white flesh trait was dominant over both red and yellow flesh traits. Using fine mapping approach, we narrowed down the white flesh gene to an 819-kb region and identified a 4906 bp sequence deletion near the *PSY1* gene. This is likely the causal variant for white flesh. Our findings provide valuable genetic resources and insight into the structural variations associated with tomato coloration.

## 2 Materials and methods

### 2.1 Plant materials and phenotyping

A white commercial hybrid variety, BaiFei (BF), was obtained from a seed market and used as a genetic resource for white fruit traits. The hybrid seeds ZheYingFen (ZYF) and JinGuan16 were used as genetic resources for red and yellow fruit traits, respectively. Four parent hybrids (F_1_) were generated by crossing BF with ZYF or JG16. F_2_ populations were produced by self-crossing the four parent hybrids, whereas F_3_ populations resulted from self-crossing F_2_ materials. Most experiments were performed under greenhouse conditions at JiangShan Farm, Weimeng Seed Co., Ltd. (Ningbo, Zhejiang Province, China). Tomato flesh color was observed after exocarp peeling. The pedigree diagram for the hybrid materials used in this study is provided in **Supplementary Figure 1**.

### 2.2 Detection of carotenoids

Carotenoid extraction and determination by high-performance liquid chromatography (HPLC) was performed referring to the previously described protocol (Lu et al., 2018). The methods of carotenoid detection on Carotenoid content was analyzed using Metware (http://www.metware.cn/) using the triple quadrupole-linear ion trap (QTRAP^®^) 6500+ liquid chromatography-tandem mass spectrometry (LC-MS/MS) platform (AB Sciex, Toronto, ON, Canada). Approximately 50 mg of freeze-dried ground powder was sampled and extracted with a 0.5 mL mixed solution of n-hexane, acetone, and ethanol (1:1:1, v/v/v). The sample was vortexed for 20 min at room temperature, and supernatants were collected after centrifugation at 12,000 r/min for 5 min at 4°C. The residue was subjected to a second extraction using the same procedure, dried, and reconstituted in 100 μL of dichloromethane. The resulting solution was filtered through a 0.22 μm membrane filter for LC-MS/MS analysis. Sample extracts were analyzed using an ultraperformance LC-atmospheric-pressure chemical ionization-tandem mass spectrometry (UPLC-APCI-MS/MS) system, with UPLC performed using an ExionLC™ AD device and MS performed using a model 6500 Triple Quadrupole instrument (Applied Biosystems, Waltham, MA, USA). The experimental parameters were set as follows. For the LC system, a YMC C30 column (3 μm, 100 mm × 2.0 mm) was used, with a solvent system of methanol and acetonitrile (1:3, v/v) containing 0.01% butylated hydroxytoluene (BHT) and 0.1% formic acid (A), and methyl tert-butyl ether with 0.01% BHT (B). The gradient program was 0% B from 0 to 3 min, increased to 70% B from 3 to 5 min, further increased to 95% B from 5 to 9 min, and returned to 0% B from 10 to 11 min. The flow rate was maintained at 0.8 mL/min, column temperature at 28°C, and injection volume at 2 μL. Detection was performed using both linear ion trap and triple quadrupole scans on the aforementioned QTRAP^®^ 6500+ LC-MS/MS System equipped with an APCI Heated Nebulizer, operating in positive ion mode and controlled by Analyst 1.6.3 software (AB Sciex). The APCI source operated with the ion source set to APCI+, source temperature of 350°C, and curtain gas (CUR) set to 25.0 psi. Carotenoids were analyzed using scheduled multiple reaction monitoring (MRM), and data acquisition was conducted using Analyst 1.6.3 software (AB Sciex). Quantification of all metabolites was performed using Multiquant 3.0.3 software (AB Sciex). MS parameters, including declustering potential (DP) and collision energy (CE) for individual MRM transitions, were optimized through further adjustments of DP and CE. A specific set of MRM transitions was monitored for each period based on the elution of metabolites.

### 2.3 Bulk segregant RNA sequencing analysis

RNA was extracted from mature fruit flesh using the RNAprep Pure Extraction Kit (Tiangen Biotech, Beijing, China) according to the manufacturer’s recommendations. Two pools were created, the W1-pool and Y-pool, representing white and yellow fruit flesh samples, respectively, by mixing 20 individuals each from the BF*JG16 populations. Similarly, the W2-pool and R-pool representing the white and red fruit flesh samples, respectively, were constructed from the BF*ZYF populations. RNA sequencing generated 150 bp paired-end reads with an insert size of approximately 350 bp. Sequencing libraries were prepared using the Truseq Nano DNA HT Sample Preparation Kit (Illumina, San Diego, CA, USA) and sequenced on the Illumina HiSeq4000 platform. The quality of sequencing data was assessed using FastQC (https://github.com/s-andrews/FastQC/), followed by filtering of low-quality reads. The Mutation Mapping Analysis Pipeline for Pooled RNA-seq (MMAPPR) pipeline was used for BSR (Hill et al., 2013).

### 2.4 Fine mapping

Genotyping was performed using competitive allele-specific PCR assays based on kompetitive allele-specific PCR (KASP™) technology (LGC Genomics, Teddington, Middlesex, UK) at LGC Genomics. Single nucleotide polymorphisms (SNPs) detected in the candidate region were selected to design KASP markers (**Supplementary Table 5**). The KASP assay mix was blended with 100 ng/µL FAM, 100 ng/µL HEX, 100 ng/µL R, and distilled deionized water, with a volume ratio of 12:12:30:46. The KASP reaction was performed in a reaction volume of 5.07 µL, with 2.5 µL DNA, 2.5 µL KASP master mix and 0.07 µL KASP assay mix. The KASP protocol comprised the stage 1 preread stage of 30°C for 1 min; stage 2 hold stage of 94°C for 15 min; stage 3 PCR stage (touchdown) for 94°C for 20 s then 61°C for 1 min (decrease of 0.6°C), recycling nine times (a total of 10 cycles), achieving a final annealing temperature of 55°C; stage 4 PCR stage of 94°C for 20 s, 55°C for 1 min, and recycling 26 times; and stage 5 postread stage of 30°C for 1 min. After amplification, an LGC Omega F reader was used to detect the fluorescence signal and validate the classification. Further cycles were conducted if the genotyping was insufficient, and the results were validated.

### 2.5 Cloning and RT-qPCR analysis of candidate gene

F_4_ individuals (BF*ZYF-8-2-1·and·BF*ZYF-11-1-8) exhibiting pure white or yellow phenotypes were used to amplify the candidate gene and conduct gene expression analysis. PCR amplification was performed using KOD ONE PCR Master Mix (Toyobo, Osaka, Japan). Gene expression levels at the breaker and mature stages were examined by RT-qPCR. Total RNA was isolated using the RNAprep Pure Extraction Kit (Tiangen), assessed for quality by 1% agarose gel electrophoresis, and converted to cDNA using HiScript II Q RT SuperMix for qPCR (+gDNA wiper) (Cat no. R223, Vazyme, Shanghai, China). PCR was performed using the ChamQ Universal SYBR qPCR Master Mix (Cat No. Q711-02, Toyobo), on a CFX96 Touch Real-Time PCR Detection System (Bio-Rad, Hercules, CA, USA). The tomato actin gene was used as an internal control and all analyses were performed with three biological and technical replicates. All primers used for RT-qPCR are listed in **Supplementary Table 6**.

## 3 Results

### 3.1 Difference in metabolites between white and yellow fruit flesh in tomatoes

**Figure 1:**
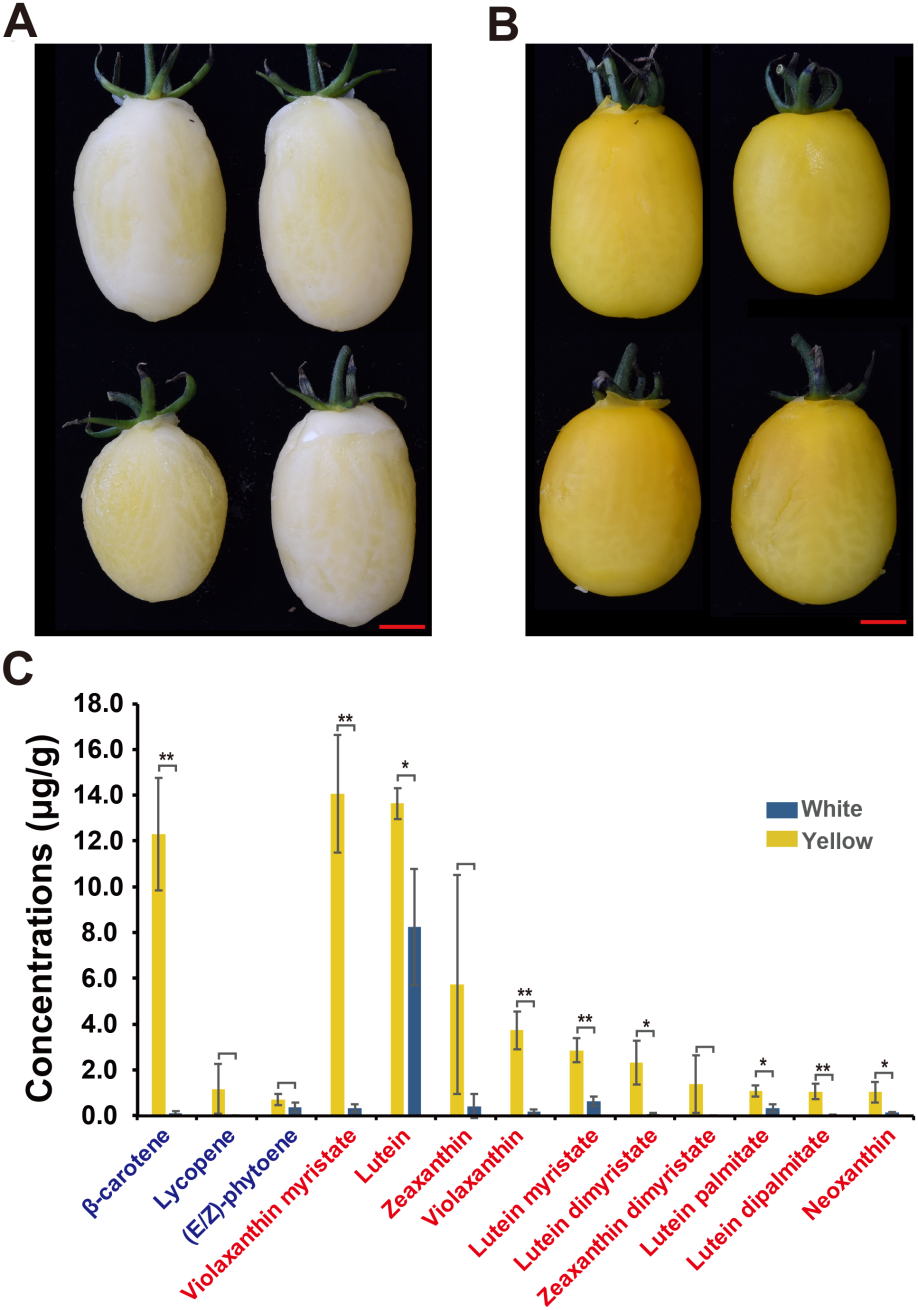
Tomato fruit color phenotypes and carotenoid concentrations in white and yellow flesh fruits. **(A, B)** Illustrations of **(A)** white (BF*ZYF-8-2-1) and **(B)** yellow (BF*ZYF-11-1-8) flesh fruit colors. **(C)** Carotenoid concentrations in white and yellow flesh fruits. Carotenes are marked in blue, and xanthophylls are marked in red. Student’s *t*-test, **P<0.01, *P<0.05.

Carotenoids are known for their antioxidant, anticancer, and vision-supporting properties and typically impart orange, red, and yellow colors to flowers and fruits (Liu et al., 2015). To investigate the phenotypic differences between white- and yellow-flesh tomato fruits, two F_4_ populations, BF*ZYF-8-2-1 (Yellowish white flesh, number 155D, Horticulture Royal Society Chart) and BF*ZYF-11-1-8 (Vivid yellow flesh, number 9B, Horticulture Royal Society Chart), were selected to assess carotenoid variation (**Figures 1A, B**). Carotenoids can be categorized into two main groups: xanthophylls and carotenes (Liu et al., 2015). In yellow fruits, β-carotene was identified as the predominant carotene, which was significantly higher compared to white flesh fruits (**Figure 1C**). Lycopene, typically abundant in red tomato fruits, was present in trace amounts in yellow fruits and was undetectable in white fruits. Small amounts of (E/Z)-phytoene were detected in both yellow and white fruits. Regarding xanthophylls, yellow fruits showed a trend of higher accumulation than white fruits (**Figure 1**; **Supplementary Table 1**), notably violaxanthin myristate, which exhibited significant differences between the groups. Overall, white fruits accumulated fewer carotenoids than yellow fruits.

### 3.2 Inheritance pattern of white fruit color in tomatoes

To explore the inheritance pattern of white fruit color in tomatoes, two sets of F_3_ populations derived from the four parent hybrids were analyzed. For white and yellow traits, four F_3_ populations initially displayed white flesh phenotypes, with subsequent observations revealing 50 yellow and 143 white fruit offspring (**Table 1**). The ratio observed fits a Mendelian ratio (χ^2^ = 0.694, P = 0.45), indicating that white fruit color is dominant to yellow in this population. Similarly, for the white and red flesh traits, eight out of 16 populations, starting with a white phenotype, exhibited pure white fruit phenotypes. The remaining populations comprised 89 red and 217 white individuals (**Table 1**), fitting a Mendelian ratio (χ2 = 2.7233, P = 0.099), suggesting the dominance of white fruit color over red in this population.

**Table 1:** Phenotypes of segregation populations.

Population Code	Genealogical information	Pre-generationphenotype	Phenotype		
			Yellow	Red	White
XT-3	BF*HG16-1-1	white	12	/	36
XT-4	BF*HG16-1-4	white	11	/	38
XT-5	BF*HG16-3-7	white	16	/	31
XT-6	BF*HG16-6-2	white	14	/	34
XT-40	BF*ZYF-8-1	white	/	0	40
XT-41	BF*ZYF-8-2	white	/	0	40
XT-42	BF*ZYF-8-3	white	/	0	40
XT-43	BF*ZYF-11-1	white	/	13	22
XT-44	BF*ZYF-11-2	white	/	11	27
XT-45	BF*ZYF-11-3	white	/	0	40
XT-46	BF*ZYF-12-1	white	/	0	40
XT-50	BF*ZYF-20-1	white	/	14	25
XT-51	BF*ZYF-20-2	white	/	0	40
XT-52	BF*ZYF-22-1	white	/	13	27
XT-53	BF*ZYF-22-2	white	/	9	29
XT-54	BF*ZYF-24-1	white	/	12	27
XT-55	BF*ZYF-24-2	white	/	0	40
XT-56	BF*ZYF-26-1	white	/	9	29
XT-58	BF*ZYF-26-3	white	/	8	31
XT-59	BF*ZYF-26-4	white	/	0	40

* means hybridization, detail information is provided in **Supplementary Figure 1**.

### 3.3 Genetic mapping of the candidate locus for white fruit color

**Figure 2:**
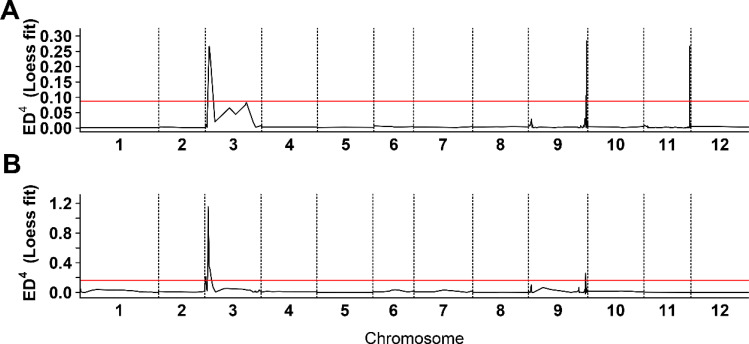
Bulk segregant RNA-seq analysis of white flesh in tomato. **(A)** Distribution of Euclidean distance (ED) association values across chromosomes in BF*HG16 population. **(B)** Distribution of ED association values across chromosomes in BF*ZYF population. The x-axis denotes chromosome names, the black line represents fitted ED values, and the red line indicates the significance association threshold. Higher ED values indicate stronger single nucleotide polymorphisms locus associations.

To identify candidate loci associated with white fruit color in tomatoes, BSR analysis was performed. Equal quantities of RNA from the flesh of 20 white and 20 yellow tomatoes from the BF*HG16 population were pooled to generate White1_pool and Yellow_pool, respectively. Similarly, RNA from 20 white and 20 red tomatoes from the BF*ZYF population was pooled to create the White2_pool and Red_pool. Sequencing of these four pools generated 25.05 Gb of raw data, with Q20 > 98.11%, Q30 > 94.45%, and guanine-cytosine ratios ranging from 42.76% to 43.13% (**Supplementary Table 2**). A BSR analysis pipeline was employed to identify candidate regions. For the White1_pool and yellow pool, three significant peaks above the threshold were detected (**Figure 2A**; **Supplementary Table 3**). Similarly, the White2_pool and red pool showed four significant peaks above the threshold (**Figure 2B**). Chromosome 3 showed the most significant peak in both analyses, with an overlapping region (SL4.0ch03:3665098-6832118) identified as a candidate region.

### 3.4 4906-bp sequence absence near *PSY1* confers white fruit trait

**Figure 3:**
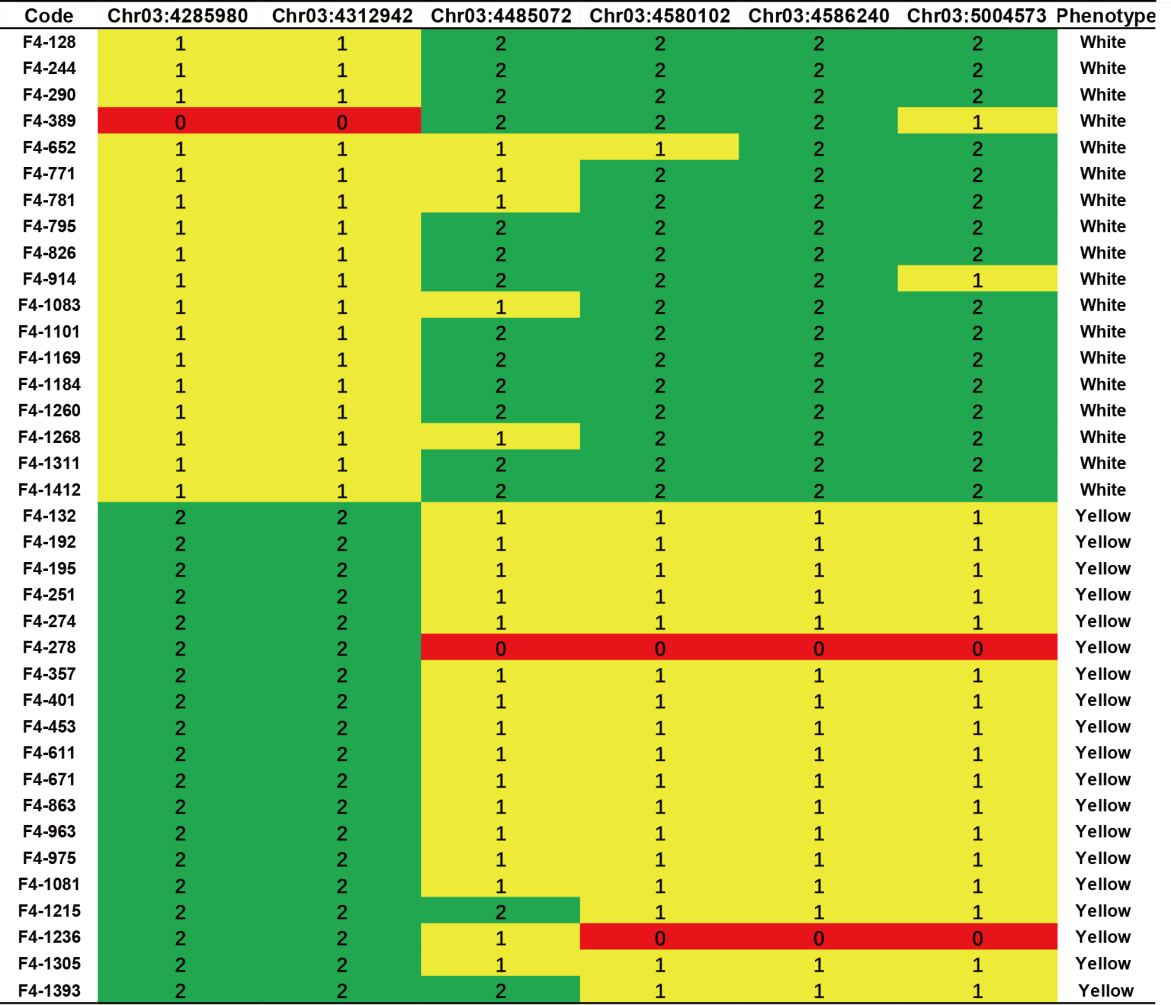
Fine mapping and genotyping results of the candidate region. Genotyping result of six KASP markers in typical F_4_ recombinant lines. Green indicates homozygous white genotypes, red indicates homozygous yellow genotypes, and yellow indicates heterozygous regions.

To validate this candidate region, we mapped RNA-seq reads to the reference genome and identified variations that served as a source for SNP marker selection. Two markers, Chr03:4312942 and Chr03:4485072, showed genotype segregation and were used to genotype F_3_ populations. Strong correlations between genotype and phenotype were observed in the XT-5 population from the BF*HG16 offspring and in XT-54, XT-56, and XT-56 from the BF*ZYF offspring (**Supplementary Table 4**). We then selected offspring from the heterozygous lines in the XT-5 population for further fine mapping. Four markers (Chr03:4312942, Chr03:4485072, Chr03:4580102, and Chr03:4586240; details in **Supplementary Table 5**) exhibited genotype segregation in XT-3 populations and were used to screen recombinants. After screening approximately 1500 F_4_ individuals, we identified 37 recombinants with pure white or heterozygous genotypes, refining the candidate genes to an 819 kb region (SL4.0ch03:3665098-4485072) based on recombinant genotypes and phenotypes (**Figure 3**).

**Figure 4:**
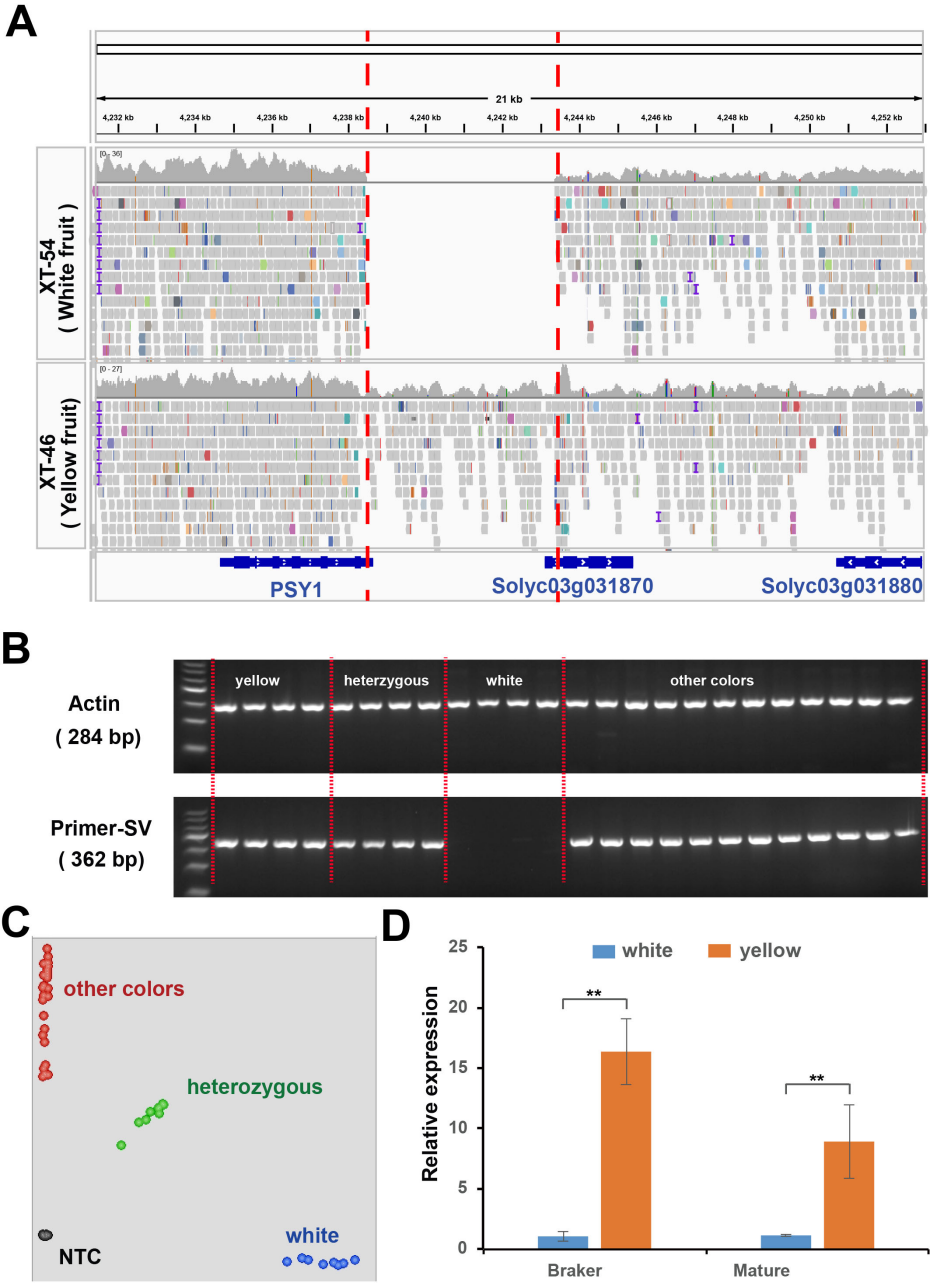
Structure variations related to white flesh phenotypes. **(A)** Re-sequencing results of pure white and pure yellow genotypes in the 21 kb candidate region near *PSY1*. Colored squares indicate the re-sequencing results, with gene structures depicted in blue. **(B)** PCR analysis of the co-dominant marker InDel-White in the parental lines. **(C)** KASP analysis of the single nucleotide polymorphism adjacent to the absent fragments. **(D)** Relative expression of *PSY1* in homozygous white and yellow individuals. Student’s t-test, ** P<0.01.

To identify the causal gene for white flesh formation in tomatoes, we resequenced F_4_ individuals (BF*ZYF-8-2-1 and BF*ZYF-11-1-8) with pure white or yellow phenotypes. A 4906-bp sequence was absent near *PSY1* in whites. The deletion results in the absence of the 3’ untranslated region (UTR) of *PSY1* and the initiation codon of *SolyC03g031870* (**Figure 4A**), which encodes an Acyl-CoA synthetase that is crucial for pollen development, male fertility, and thermotolerance (Hu et al., 2020; Xie et al., 2024). Gel markers designed for genotyping confirmed that the deletion was exclusively detected in white flesh germplasm (**Figure 4B**; **Supplementary Table 6**), and a nearby SNP marker (Chr3:4285980; **Figure 4C**) further validated this finding. Gene expression analysis revealed significantly higher *PSY1* expression in yellow flesh than in white fruit (**Figure 4D**), whereas *SolyC03g031870* expression was undetectable in both varieties. Therefore, we conclude that this deletion likely affects the expression of *PSY1*, leading to the formation of white flesh.

To explore whether the variability in flesh color across natural populations can be explained by this gene, we examined the re-sequencing results from accessions of various colors. Remarkably, causal deletions were only found in white fruit varieties based on read coverage in this region (**Supplementary Table 7**), which was corroborated by structural variation and nearby SNP markers (**Figure 4D**). These findings highlight that the deletion is the causal variant in white flesh formation. In addition, we developed a KASP marker that was close to the deletion. The genotyping results confirmed that the GG genotype was exclusive to white varieties (**Figure 4D**), suggesting its utility in further molecular breeding.

## 4 Discussion

Carotenoids are essential pigments synthesized by plants. These pigments enrich the vibrant spectrum of color found in fruits (Khoo et al., 2011). The presence and distribution of carotenoids within fruit tissues are fundamental determinants of fruit coloration and significantly influence consumer preference. β-carotene, lycopene, and lutein are among the key carotenoids responsible for infusing fruits with their characteristic yellow, orange, and red hues, respectively (Saini et al., 2015). Synthesized primarily within chromoplasts during fruit ripening via a sequence of enzymatic reactions, carotenoid biosynthesis is orchestrated by pivotal genes that include *PSY* (Yang et al., 2023). The tomato genome possesses three *PSY* genes, *PSY1*, *PSY2*, and *PSY3*, each exhibiting distinct tissue-specific expression patterns, predominantly in the fruit, petals, and roots (Fraser et al., 2001, 2007). Throughout tomato ripening, the expression of *PSY1* closely correlates with the accumulation of carotenoids, which in turn influences the final coloration of the fruit (Ray et al., 1992; Cao et al., 2023). Reductions in total carotenoid content are observed in fruits with yellow flesh when *PSY1* is subjected to antisense silencing, gene editing or knock-out mutations (Ray et al., 1992; Cao et al., 2023; Yang et al., 2023). Conversely, overexpression of this gene leads to increased levels of β-carotene (Fraser et al., 2007).

Similar findings regarding the correlation between upregulated *PSY* gene expression and heightened carotenoid accumulation have been documented across various fruits and thus resulted in color changes, including pepper (Rodriguez-Uribe et al., 2012), citrus (Peng et al., 2013), loquat (Xiumin et al., 2014), watermelon (Pin et al., 2015), and raspberry (Mizuno et al., 2017). For example, *ClPSY1* plays a dominant role in carotenoid biosynthesis during the ripening of watermelon fruit, with its expression significantly lower in the white-fleshed variety compared to the pigmented fruits in watermelon (Fang et al., 2022), Similarly, *ZmPSY1* plays a crucial role in carotenogenesis within the endosperm (Li et al., 2008). Furthermore, the activation of the native *PSY1* promoter has been shown to induce carotenoid biosynthesis in embryogenic rice callus (Sobrino-Mengual et al., 2024).

Tomatoes have served as a prominent model for studying carotenoid metabolism, revealing a spectrum of phenotypes associated with various *PSY1* variations, such as yellow, tangerine, white, and bicolor phenotypes (Fray and Grierson, 1993; Pereira-Castro and Moreira, 2021; Bulot et al., 2022). A 3789-bp deletion in the *PSY1* promoter has been identified as the causal variation for the bicolor phenotype in tomatoes (Bulot et al., 2022), whereas a duplication and inversion involving *PSY1* and an adjacent gene has been verified as the causal variation in the *r^y^* phenotype (Bulot et al., 2022). In addition, the insertion of a single long terminal repeat from the Rider transposon in the first exon of *PSY1* resulted in a non-functional protein, which was identified as the determinant of yellow cultivars (Fray and Grierson, 1993). Mutants *r^2997^* and *r^3576^* in the *PSY1* gene caused yellow flesh phenotypes, whereas the double mutant *r^3576^/t^3406^* exhibits a typical yellow flesh phenotype, and the double mutant *r^2997^/t^3406^* shows tangerine phenotypes (Kachanovsky et al., 2012). The 3’-UTR is a critical region, harboring cis-elements that affect an mRNA’s processing, localization, translation, and stability, is pivotal in gene regulation and could act as regulatory hubs for the environmental control of gene expression (Pereira-Castro and Moreira, 2021; Emma and Martin, 2024). For instance, a T-DNA insertion in the 3’-UTR of SlFAF1/2c results in a gain-of-function mutation that disrupts the regulatory function of the 3’-UTR, leading to increased expression of SlFAF1/2c in tomato (Zhang et al., 2023). Moreover, research on rice revealed that a stowaway-like MITE (sMITE) embedded in the 3’-UTR of OsGhd2 repress the gene expression and thus affected the grain number, plant height, and heading date (Shen et al., 2017). Here, we identified a 4906-bp sequence absence at the 3’ end of the *PSY1* gene in white flesh in tomato, which might be responsible for the white flesh phenotype. The decreased expression of *PSY1* in white fruits (**Figure 4**) provides additional supporting evidence. Thus, it is reasonable to speculate that this deletion may be a causal factor for white flesh formation.

Our study identified a novel variation of *PSY1* underlying the unique white flesh coloration observed in tomatoes. Specifically, structural variation involving a 4906-bp fragment near *PSY1*, which significantly correlates with the manifestation of white flesh phenotypes, was absent in white flesh tomato germplasms. These findings offer valuable insights into the molecular pathways governing fruit coloration and facilitate advancements in the molecular breeding of white flesh tomato varieties.

## Data Availability

The datasets presented in this study can be found in online repositories. The names of the repository/repositories and accession number(s) can be found in the article/**Supplementary Material**.
