# Supplementary figures and images for: Genetic mapping and molecular marker development for white flesh color in tomato
Source: Front Plant Sci. 2024 Sep 3;15:1459013. doi: 10.3389/fpls.2024.1459013 (PMC11405233; doi:10.3389/fpls.2024.1459013)

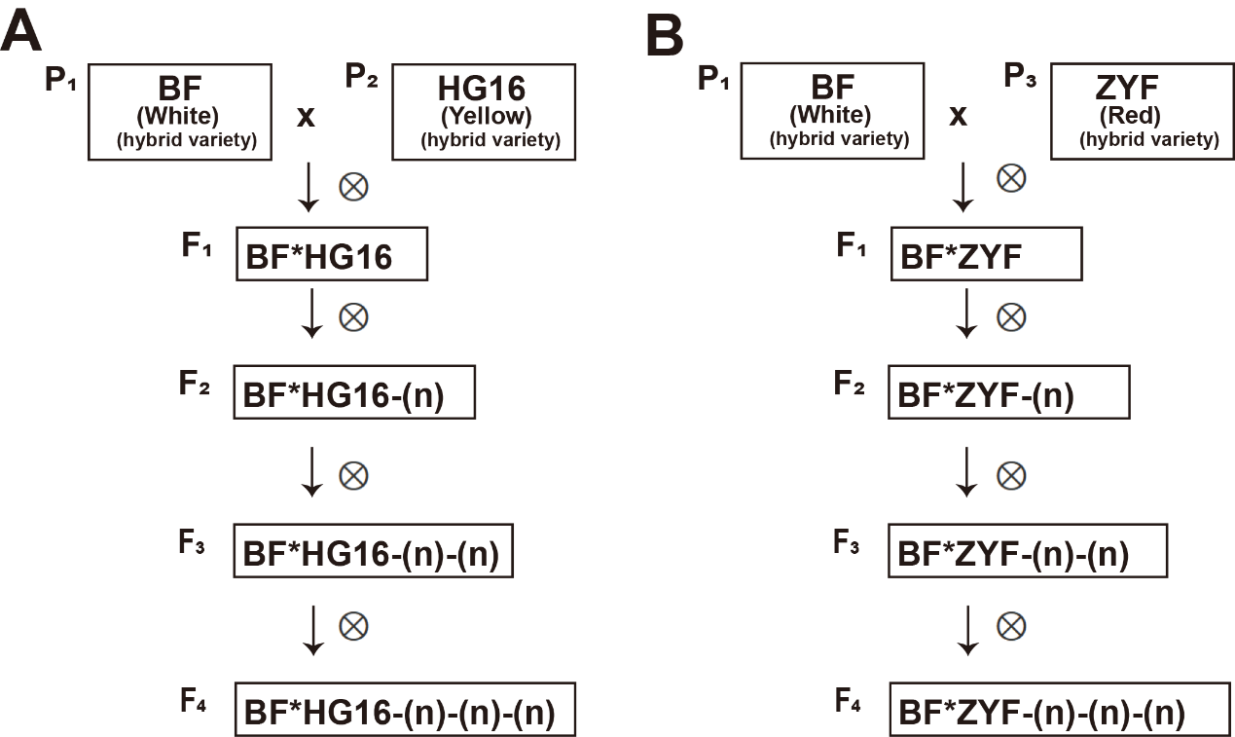


**Supplementary Figure 1** The pedigree diagram for the hybrid materials used in this study.

Supplement: Supplementary file 1 [file DataSheet1.docx]
